# Rare pulmonary barotrauma after explosive decompression: a case report

**DOI:** 10.1186/s12890-020-01321-5

**Published:** 2020-11-09

**Authors:** Jakub Tlapák, Boris Oniščenko, Petr Došel, Pavel Požár, Petr Chmátal, Michal Hájek

**Affiliations:** 1 The Institute of Aviation Medicine, Prague, Czech Republic; 2 Department of Military Surgery, Faculty of Military Health Sciences, University of Defence, Hradec Kralove, Czech Republic; 3 Third Faculty of Medicine, Charles University in Prague, Prague, Czech Republic; 4 Center of Hyperbaric Medicine, Ostrava City Hospital, Ostrava, Czech Republic; 5 Department of Biomedical Sciences, Faculty of Medicine, University of Ostrava, Ostrava, Czech Republic

**Keywords:** Case report, Pneumothorax, Explosive decompression, Pulmonary barotrauma

## Abstract

**Background:**

Pneumothorax as a consequence of pulmonary barotrauma during explosive decompression military crew training in a hypobaric chamber is an extremely rare and sparsely diagnosed complication. Extensive bilateral tissue damage is even more unexpected.

**Case presentation:**

A 26-year-old active duty Air Force pilot was performing an explosive decompression simulation from 8000 ft. (2438.4 m) to 25,000 ft. (7620 m) in a 1.5 s interval. The training was interrupted due to the pilot’s apparent health complications. After transfer to the emergency department, a CT scan showed bilateral lung barotrauma with emphysema.

**Conclusions:**

The case report shows extensive emphysema and pneumothorax after a rapid decompression done for training purposes. It is a possible but rare complication. The cause remains unclear, with suspicion of a predisposed lung disease.

## Background

Humans exposed to pressure changes may suffer from barotraumas – tissue damage in gas-filled cavities inside the body [1]. Barotraumas are dependent on several factors such as the magnitude of the pressure change, the rate of change, and others [2]. They may be common while diving or in aviation. During pressure changes within the atmosphere, typical barotraumas occur in the middle ear cavity, sinuses, and sparingly in the gastrointestinal tract, but lung barotraumas are very rare, as will be discussed. Lung barotrauma occurs during a decrease in pressure, when the expanding gas from the lungs cannot be ventilated sufficiently. This causes an increase intrathoracic pressure which at some point will cause structural damage – pneumothorax. There are some reports describing a development of pneumothorax in aviation. A few cases were reported [3, 4], but they were classified as idiopathic and thus could not be automatically considered as barotraumas. An increased risk of flight-related pneumothorax is also found in humans who are predisposed to conditions such as Birt-Hogg-Dubé syndrome, alpha-1-antitrypsin deficiency and congenital pulmonary airway malformation/congenital cystic adenomatoid malformation [5, 6].

Very quick decompression, also known as explosive decompression, is a flight risk connected to pressurized aircraft cabins. Military personnel usually train for this situation according to various aviation medicine training schedules, such as the NATO standard procedure – STANAG 3114. This training is done periodically throughout the world and it is required for all fast jet pilots. This means countless of exposures, with no – or unpublished – complications. Some of these studies describe problems encountered during training expositions but none of them include barotrauma of the lungs. The most common problem was typically ear pain; The Japan Air Self-Defense Force gathered 17,935 exposures from 740 patients, in which 4.1% reported experiencing ear pain [7]. A retrospective study of the Italian Air Force reported incidence of ear pain in 1.5% of the 1241 participants [8]. Notably, pneumothorax did not occur in either case. In Table 1, a list of documented lung barotraumas during altitude chamber training is shown [9–12]. Our institute trains pilots as well. Over the past 10 years, we have performed a total of 566 explosive decompressions in our hypobaric chamber, with subjects from eight different countries. Three hundred ninety one of the explosive decompressions were performed from an altitude of 8000 ft. (2438.4 m) to 25,000 ft. (7620 m), 175 were from an altitude of 23,000 ft. (7010.4 m) to 43,000 (13,106.4 m) ft. and in combination with positive pressure breathing. In both profiles, the duration of the pressure change was under 2 seconds. The occurrence of the first pulmonary barotrauma is mentioned in this case report. Our current incidence rate is 0.2% in 10 years.

**Table 1:** Cases of pulmonary barotrauma during rapid decompression training in hypobaric chamber. PTX, pneumothorax

Study/Year	Type of pulmonary barotrauma	No. of subjects	Decompression in feet
Clark 1945 [9]	pneumomedistinum	2	from 8,000 to 31,000
Luft 1954 [10]	PTX	1	from 8,000 to 30,000
Holmstrom 1958 [11]	Pneumomediastinum, PTX, subcutaneous emphysema	2	from 8,000 to 22,000
Cable 2000 [12]	pulmonary barotrauma with cerebral arterial gas embolism	1	from 8,000 to 25,000

### Equipment and profiles

**Fig. 1:**
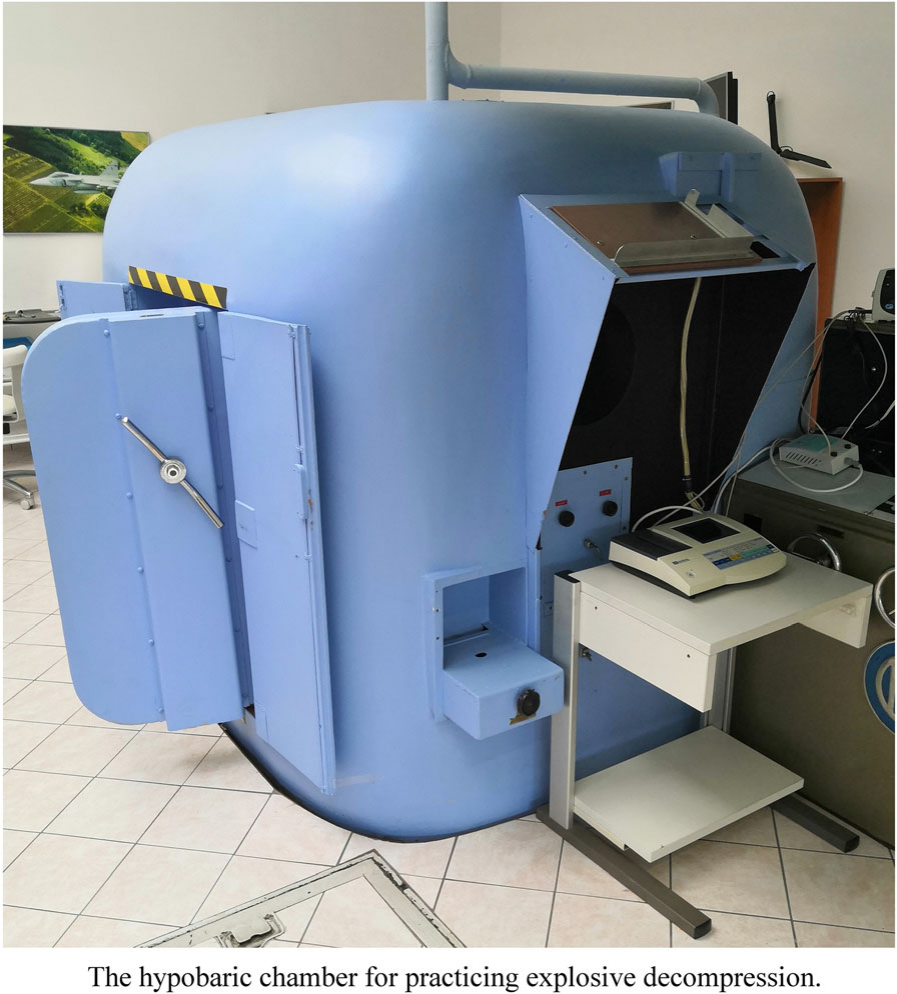
The hypobaric chamber for practicing explosive decompression

Our training program is in compliance with STANAG 3114 and starts with theoretical preparation and medical information. Instructions for the decompression are extremely important — crucial points, like the risks from holding ones breath, are emphasized on multiple occasions. The practical training takes place in the mono-place explosive decompression chamber (Fig. 1). The chamber is equipped with a KKO-5 breathing regulator. Individuals are continuously monitored by a closed-circuit television system (CCTV), ECG, heart rate monitor, and pulse oximeter. Communication is open via intercom. Besides the CCTV, the pilot is visible through a 200 mm viewing window at the height of the individual’s face and upper trunk area.

**Fig. 2:**
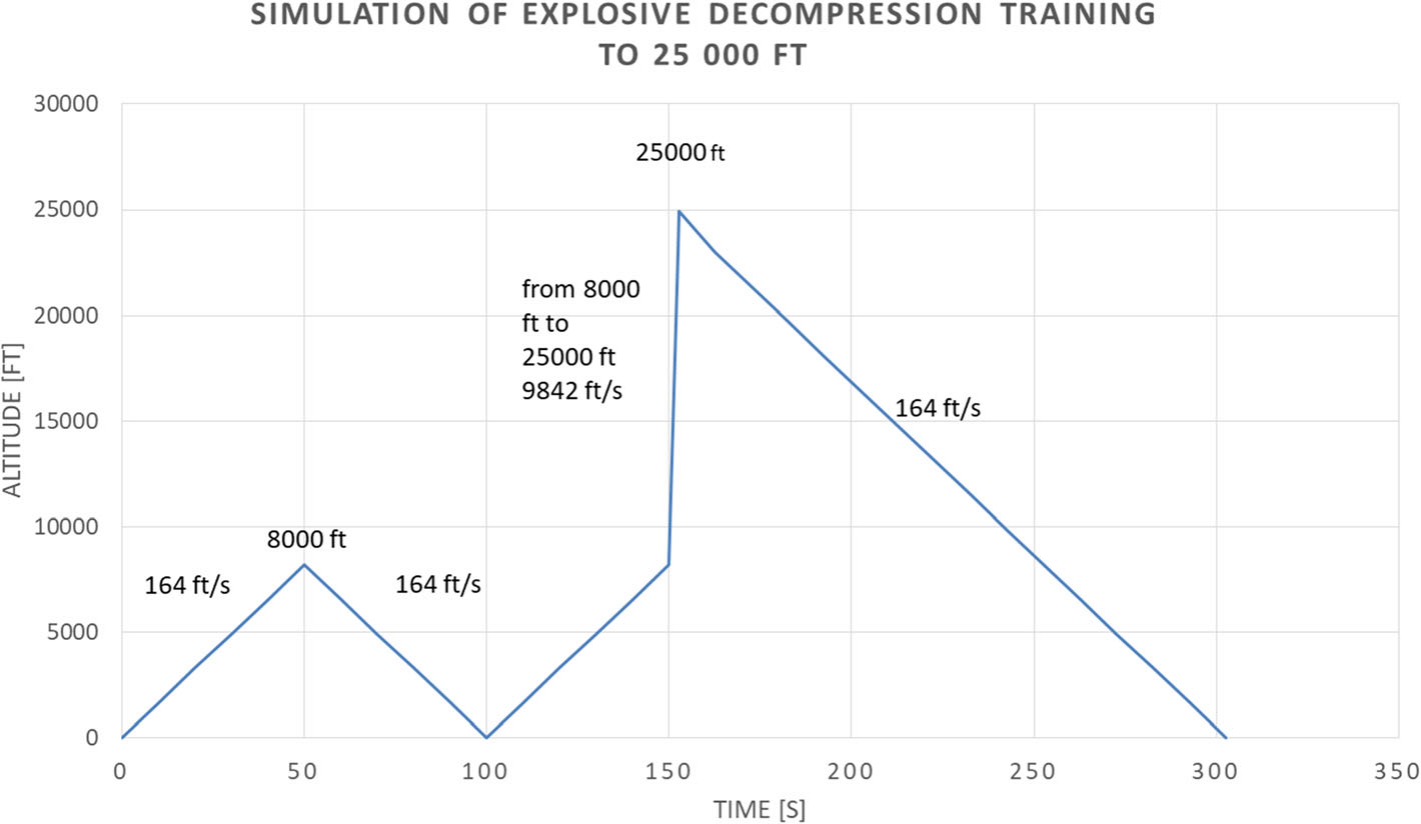
Profile of explosive decompression training: Relation of the rate of change of altitude to time. The first peak is ear and sinus check. The second peak is reached by expplosive decompression

Pilots attending the training must be healthy and on active flight status. Prior to the start of the practical training, we perform tympanometry and otoscopy, then the subject undergoes 30 min of preoxygenation, to minimize the risk of decompression sickness. The demonstration begins with a “sinus check” (paranasal sinuses barofunction test), which serves to avoid pressure equalization problems at higher altitudes and potential consequent middle ear barotraumas. Then the subject returns to the initial altitude. The instructions are repeated once again. Then, the pilot should exhale, leaving the mouth open, and run the decompression. The pressure change in this case is 278 mmHg (from 563 to 285 mmHg) and the duration is 1.5 s. This usually does not cause any problems, so the subject descends and continues with theoretical debriefing. The profile is attached in Fig. 2.

## Case presentation

The subject was a 26-year-old male Air Force pilot and a foreign state officer. The pilot had some individual flight experience, with a flight time of 120 h in the last 2 years on a L-39 Albatros, aircraft type designation. He reported no health problems before the training, nor in his medical history. He is a non-smoker and denies drug abuse. A clinical examination before the incident showed normal findings and the beginning of the practical training was normal. Problems arose the moment after the explosive decompression – the pressure inside the chamber was already stable. The pilot signaled to stop by waving his arm, than he crouched down in the seat, holding his head. Then, he produced a few grunts or cough-like sounds. He did not respond to questions about what had happened or what the problem was. After 7–8 s, an emergency descent was ordered to access the pilot. During the descent, the pilot started to respond normally. This emergency descent was likely the reason for the mild iatrogenic barotrauma of the middle ear. This issue was resolved in the next few days and is not connected to the purpose of this report.

Once on the ground, the pilot did not report any major symptoms at first. He said the main problem was sinus pain, that he did not feel any chest pain or have trouble with breathing. He described the coughing sounds as a verbalization of his pain and remembers us talking to him. In other words, he did not understand our concern in regards to lung trauma and did not seem anxious or worried. He also clearly stated that he did not hold his breath during the decompression. After finishing, he was eupneic, with normal hemodynamic parameters and a normal chest examination. After a few minutes, he developed retrosternal pain during deep inspiration and his tolerance of being in a horizontal position was reduced. These symptoms did not worsen, however, he was sent to our emergency department on suspicion of lung barotrauma.

**Fig. 3:**
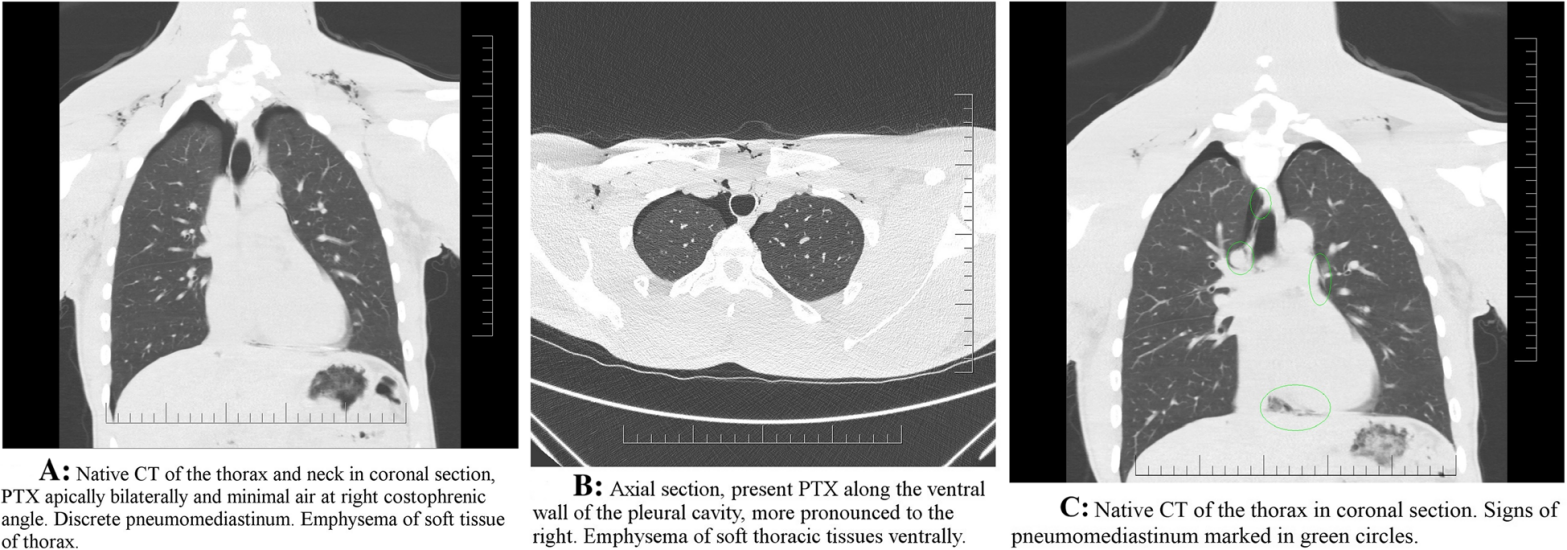
**a** Native CT of the thorax and neck in coronal section, PTX apically bilaterally and minimal air at right costophrenic angle. Discrete pneumomediastinum. Emphysema of soft tissue of thorax. **b** Axial section, present PTX along the ventral wall of the pleural cavity, more pronounced to the right. Emphysema of soft thoracic tissues ventrally. **c** Native CT of the thorax in coronal section. Signs of pneumomediastinum marked in green circles

The first chest X-ray and CT showed diffuse emphysema of the superficial and deep parts of the neck, continuing to the proximal section of the ventral chest wall (Fig. 3a, b). Diffuse pneumomediastinum signs are apparent paratracheal right, around right pulmonary artery, junction of left pulmonary veins and left atrium further are present signs in right cardiophrenic angle (Fig. 3c). A small bilateral pneumothorax was found apically and basally with pneumoperitoneum. The pneumoperitoneum was concluded to have been caused by passing gas through the hiatus in the diaphragm. There was no evidence of free fluid in the chest, no dislocations of mediastinal structures, no traumatic skeletal changes. A bronchoscopy was recommended for suspected airway injuries, but the patient refused. Conservative protocol without thoracic drainage was followed during his hospital stay. A two-day interval chest CT scan showed regression of the bilateral pneumothorax, regression of soft tissue emphysema, as well as pneumomediastinum regression. The patient stated he felt well and did not exhibit any additional symptoms, so he was discharged. After the discharge, follow-up examinations were recommended and a 2 month no-fly period was ordered. However, after 3 days, the patient left to his homeland and a contact at required medical level was lost. Pilot reported that he is doing well, without any problems and will be fit-to-fly soon.

A report done by the technical staff ruled out technical malfunction. A breathing mask was used before and after the incident without problem. All of the equipment used had required official certifications. There were not any suggestions of problems with the used equipment.

## Discussion

As stated before, lung barotrauma is very rare in aviation training, as well as during real flight incidents. During training, breathing technique must be conducted properly, and this was confirmed by the pilot. But it cannot be judged based on CCTV alone — we cannot measure intrathoracic pressure and we cannot see the trainee’s mouth due to the breathing mask. There was suspicion of simultaneous inhalation during the moment of decompression, according to the breathing regulator and lung movement in the chest. But even with initially empty lungs and with poor breathing technique, the inspiratory reserve volume should have had the ability to protect the lungs and this type of excessive damage was unexpected. There is information about possible human tolerance to rapid decompressions; this topic was extensively reviewed in Human Pulmonary Tolerance to Dynamic Over-Pressure study [13], but in conclusion, they could not state exact levels of tolerance due to limited data availability. However, the authors suggest that the dynamic overpressure, spread over 1.5 s (our case), should be tolerable for a pressure change somewhere between 375 and 675 mmHg, which is roughly two to three times more than our profile.

Similar limits are reported by Ernsting’s Aviation and Space Medicine Handbook [14]. A decompression from 8000 ft., with lungs filled to half of the total capacity, should be tolerable with a limit up to 29,700 ft., even with a shorter decompression time. Both of these sources show a sufficient safety margin in comparison to our decompression profile, although the limits where a barotrauma would occur are still unclear and probably individual.

We can speculate about a predisposed lung disease, but the pilot refused to investigate this etiology. The attending doctor recommended a bronchoscopy, as there was suspicion of bronchial damage rather than lung barotrauma. A severe case of emphysema could suggest this, but the pilot refused any invasive examinations. Some data for civil aviation were published showing an incidence up to 2% in predisposed lung diseases (cystic lung diseases in particular) [15]. However, incidence data for military pilots is not available. Physical suitability is an important factor in the selection and classification of candidates for flight training programs [16]. We couldn’t find or diagnose any predisposed lung disease, but we would suggest this as a very probable explanation. A limited idea of possible expected lung damage is reported in the cadavers study; it is stated that the presence of basal adhesions predisposes some to pulmonary barotrauma [17]. Although the conditions are different, we can compare some of the results of overpressure on human lungs.

Findings of pneumomediastinum may lead us to extend this event to also as an example of spontaneous pneumomediastinum. This uncommon problem has a direct pathophysiological connection to barotrauma of the lungs [18]. However, even if we cannot be sure about the etiology of pneumomediastinum itself, there is a clear etiology of barotrauma and this event probably couldn’t be considered spontaneous.

The available, relevant information shows a rather unexpected result; if we agree that barotrauma is possible and probable, there is still the question of the extent of lung damage with bilateral pathology and subcutaneous emphysema. We could not finish all of the suggested and requested examinations, but we can speculate about a predisposed condition which has not been revealed by any examinations of the pilot during his career. From another point of view, this incident favors the benefits of the training to show a hidden, dangerous health problem.

## Data Availability

All data discussed in the manuscript are included within this published article. Complete CT scans are available on request.

## Abbreviations

CT: Computed tomography
CCTV: Closed-circuit television system
ECG: Electrocardiogram
PTX: Pneumothorax
NATO: North Atlantic Treaty Organization
STANAG: Standardization Agreement
